# Spatial Pattern, Sources Identification, and Risk Assessment of Heavy Metals in a Typical Soda Soil from Bayannur, Northwestern China

**DOI:** 10.3390/ijerph192113880

**Published:** 2022-10-25

**Authors:** Shuncun Zhang, Tao Wang, Hao Wang, Qiangqiang Kang, Qian Zhou, Bo Chen

**Affiliations:** 1 Northwest Institute of Eco-Environment and Resources, Chinese Academy of Sciences, Lanzhou 730000, China; 2 Gansu Salinization Field Observation and Research Station, Lanzhou 730000, China; 3 University of Chinese Academy of Sciences, Beijing 100049, China; 4 Guangxi Key Laboratory of Green Chemical Materials and Safety Technology, Beibu Gulf University, Qinzhou 515000, China

**Keywords:** metal element, toxicity, ecological risks, source, Hetao Plain

## Abstract

Soil is an important natural resource in the agricultural areas of northwest China. The heavy metal concentration and ecological risk assessments are crucial for food safety and human health. This work collected 35 surface soil samples and focused on a typical soda soil quality of the Hetao Plain in Bayannur, which is an important grain production base in northern China. The concentration and composition of heavy metal (arsenic (As), cobalt (Co), copper (Cu), lead (Pb), cadmium (Cd), chromium (Cr), mercury (Hg), nickel (Ni), zinc (Zn)), soluble salts, total organic carbon (TOC), and minerals of the surface soils were analyzed to assess the biotoxicity, ecological risk, sources, and influencing factors of heavy metals in these soda soil from this region. The enrichment factors (*EF*) showed that As, Co, Cu, and Pb were not contaminated in these soils, while Cd, Cr, Hg, Ni, and Zn were lightly contaminated. The index of geoaccumulation (*I_geo_*) for the soda soils indicated that Co and Pb were uncontaminated, and Cr, Cd, Ni, Zn, Hg, Cu, and As were lightly contaminated. The potential ecological risk index (*RI*) indicated there were no or low risks for As, Co, Cr, Cu, Ni, Pb, and Zn. Although the concentrations of Cd and Hg in the soil were low, the two heavy metals exhibited moderate–high ecological risk because they have high biological toxicity. Cd in the soils from Hetao Plain in Bayannur is mainly exchangeable and reducible fractions. The other heavy metals in these soda soils are mainly in residue fraction, implying that their mobility is low and not easily absorbed and used by plants. Heavy metal fractions, principal component analysis (PCA), and correlation analysis showed that As, Co, Cr, Cu, and Pb were mainly from natural sources, while Ni, Cd, and Zn were mainly from anthropogenic discharge-related irrigation, fertilizers, and pesticide application, and Hg was mainly from winter snowfall in the study area. Naturally sourced metal elements have obvious sediment properties, and their adsorption by clay minerals and coupling with organic matter along with sediment transport sorting. The salinity and pH of soda soils in the study area have a highly positive correlation, hence the influence of factors on the concentrations of soil heavy metals are consistent. For anthropogenically imported heavy metals, increasing salinity and pH promote the precipitation of metallic elements in water. Cd is present as an exchangeable and reducible fraction, while Ni and Zn are mainly sequestered by organic matter and clay minerals.

## 1. Introduction

Soil is not only a natural resource for human survival but one of the most important components of the ecosystem. However, the heavy metal pollution that accompanies economic development and the high-intensity agricultural activities of modern society has become a serious problem worldwide [1,2]. Although many metals are essential elements for plant life, they can be harmful to humans, animals, plants, and microorganisms when their concentration exceeds biological tolerance. High concentrations of heavy metals in living tissues can lead to severe organ damage, neurological disorders, and ultimately death [3]. High levels of heavy metals in soils can reduce the abundance, diversity, and activity of microorganisms [4,5]. Heavy metals in soils come from the weathering of soil-forming material and various anthropogenic contributions. Soil contamination with heavy metals lead (Pb), zinc (Zn), cadmium (Cd), and copper (Cu) in urban areas generally originates from traffic, paint, and many other industrial products [6]. The heavy metal of agricultural soils is often influenced by the matrix of soil formation, which is closely related to soil grain size, specific area, and consolidation processes over time [7]. Silt and clay in minerals have a particularly important influence on the transport and storage of heavy metals in river sediments. Montmorillonite and illite are the main components of clay minerals in sediments and in suspended particulate matter, and soils or sediments containing montmorillonite clays are considered good sorbents due to the presence of different active sites such as surface and ion exchange sites [8]. However, it is also worth noting that heavy metals from fossil combustion and other sources travel long distances in the form of aerosol particles, as well as inputs from sources such as the application of organic materials and pollutants in fertilizers [9,10,11]. The chemical properties of the soil are the main determinants of the solubility, mobility, and availability of heavy metals in the soil. It has been proved that heavy metal elements can also be associated with the generation of carbonates and phosphates in an alkaline environment [12]. Since the solubility products of these compounds are relatively low, they make heavy metals accumulate in the alkaline environment [13]. Whereas, in acidic environments (such as acidic sulfate deposits), it is easy to form sulfates with high mobility [14]. E. E. Golia et al., 2019 analyzed potentially toxic elements (PTE) in soil, in Central Greece during 2013 to 2015, and found a high correlation coefficient between the soil pH and PTE concentration in Alfisols, reflecting that soil pH is the most important factor affecting PTEs [15]. Sung-Wook Yun et al., 2017 researched the transport and spatial distribution of metals in South Korean surface soil, and confirmed that the highest concentrations of As and Pb occurred in agricultural soils closest to an abandoned mine site due to climatic factors such as wind and precipitation that influenced the movement of metal-containing mine wastes [16].

At present, many agricultural lands in China are threatened by various kinds of pollution, and the area of arable land with moderate or heavy pollution is about 3.33 million hectares, and the production and ecological risks caused by soil pollution in many areas are already very serious. The exceedance rate of inorganic pollutants accounted for 82.8% of the total number of sites, while the exceedance rate of cadmium sites was 7% [17,18,19]. Li Zhiyuan et al., 2014 summarized the available data in the literature (2005–2012) on heavy metal-contaminated soils originating from mining areas in China, and argued that Cd, Pb, Cu, Zn, Hg, As, and Ni were selected as priority heavy metals for control, and tungsten, manganese, Pb-Zn, and antimony mines were selected as priority mine categories, with the southern provinces and Liaoning Province selected as priority provinces for control [20]. Qianqi Yang et al., 2018 reviewed the heavy metal concentrations in soils from 402 industrial sites and 1041 agricultural sites in China. The results showed that heavy metal pollution and associated risks were more severe due to Cd, Pb, and As. Heavy metal pollution and associated risks were more severe in industrial areas than in agricultural areas, while heavy metal pollution was more severe in southeast China than in northwest China. Cd, Pb, and As were identified as priority heavy metals for control; mining areas were priority areas for control compared to other areas in industrial areas; and food crop plantations were priority areas for control in agricultural areas [6]. The Hetao irrigation area in Bayannur City is the largest self-flowing irrigation area in Asia, one of the three major high-quality wheat-producing areas in the world, and the nationally important grain and oil production base; additionally, the largest organic raw milk, sunflower seeds, and dehydrated vegetables’ production base in China. Therefore, the assessment of heavy metal contaminants in agricultural fields and the mechanisms for their prevention are essential for the quality and safety of food and agricultural products as well as for human and animal health. However, there is little published research literature on heavy metals in agricultural soils from this area. In this paper, the concentrations of As, Cd, Co, Cr, Cu, Hg, Ni, Pb, and Zn in agricultural soils in Bayannur city were analyzed and the ecological risks were assessed using Enrichment (*EF*), Index of Geo-Accumulation (*I_geo_*), Potential Ecological Risk Index (*RI*), and Risk Assessment Code. Heavy metals, minerals, and ions were also investigated by principal component analysis and correlation analysis for heavy metal sources and influencing factors. The article aims to provide a scientific basis for the prevention and control of potential pollution of arable soils in the study area.

## 2. Materials and Methods

### 2.1. Study Area

The study area is the southern Hetao Plain in Bayannur City, Inner Mongolia Autonomous Region, China, under a typical mid-temperate continental monsoon climate, with an average annual temperature from 3.7 °C to 7.6 °C and rainfall of 130–285 mm [21,22]. The Hetao Plain is a large alluvial plain of the Yellow River, covering an area of nearly 15,900 square kilometers. This area is about 1000 m above sea level, with the Yellow River to the south and the Langshan Mountain to the north [23]. Since the Qing Dynasty, this area has been irrigated by opening canals to divert water from the Yellow River. Currently, it is the most important agricultural irrigation area and commodity grain base in the Inner Mongolia Autonomous Region.

**Figure 1:**
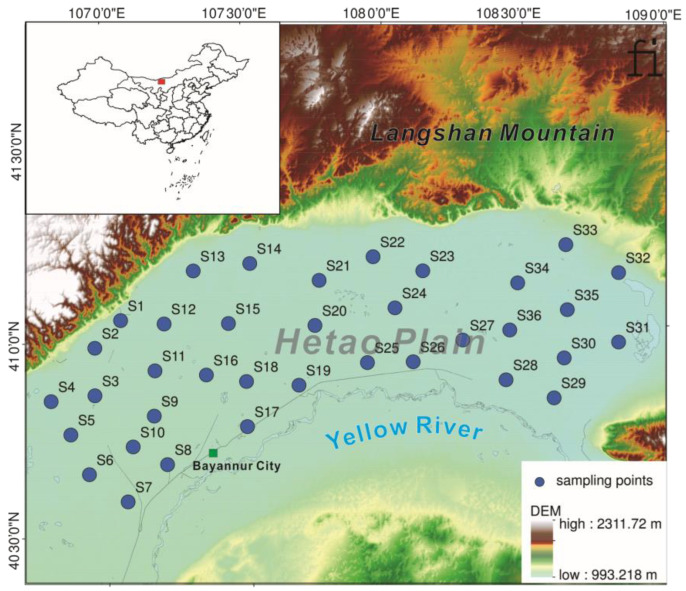
Location of the study area and sample sites.

A total of 35 surface soil (0–20 cm) samples were collected from farmland in the study area (Figure 1). The samples were collected within each grid using the five-point method: four sub-sample points were set around each central sample point, and the sub-samples were mixed with the central sample in equal amounts as the mixed sample for this site. The samples were brought to the laboratory, naturally dried, ground, passed through a 2 mm sieve to remove impurities, and then passed through a 0.149 mm sieve for testing [24].

### 2.2. Methods

#### 2.2.1. Total Organic Carbon (TOC) and Mineral Analysis

TOC was determined by an elemental analyzer (NCT ECS 8024 CN; Milano, Italy) for the organic carbon content of the samples. The samples were ground, passed through a 100 mesh sieve, and the organic carbon in the soil samples was cauterized by burning at a high temperature of 1100° to release carbon dioxide, and then the CO_2_ content was measured to calculate the TOC [25]. For mineral analysis, the samples were ground, passed through a 200-mesh sieve, and weighed about 2 g. The samples were analyzed for mineral species and content using an X-ray diffraction instrument (Rigaku Ultima IV, Tokyo, Japan). The diffraction data obtained from the samples were compared with the mine;al standard data from the International Diffraction Data Center to determine the physical appearance. The ratio of the strongest diffraction peak of the mineral in the analyzed sample to the strongest diffraction peak of the standard mineral was used to estimate the percent mineral content.

#### 2.2.2. pH, Electrical Conductivity (EC), Anions and Cations

The soil samples were air-dried, ground finely, and sieved through an 18-mesh sieve. A 2.5:1 water to soil ratio leachate was prepared, and the pH of the soil was determined using a Sartorius standard pH meter PB-10 [26]. A 5:1 water-to-soil ratio leachate was prepared and the EC of the soil solution was determined using a DDSJ-308A electrical conductivity (Raycom, Beijing, China) [27,28]; Cl^−^, ${SO}_{4}^{2-}$, ${NO}_{3}^{-}$, K^+^, Na^+^, Ca^2+^, and Mg^2+^ were determined by ion chromatography (Metrohm, Herisau, Switzerland), and ${\mathrm{CO}}_{3}^{2-}$ and ${\mathrm{HCO}}_{3}^{-}$ were determined by double indicator-neutralization titration [29].

#### 2.2.3. Major Elements, Trace Elements Measurements

The samples were weighed 20~30 mg (accurate to 0.01 mg) through a 200-mesh sieve, and the samples were digested in a PTFE digestion tank using HNO_3_-HF-HClO_4_, and after complete digestion, the digested material was transferred to a clean volumetric flask and diluted to 1000 times the weight of the samples using ultrapure water, and the determination of the major elements was performed using an inductively coupled isotope emission spectrometer (PerkinElmer Optima 8000; Waltham, MA, USA) for determination of major elements and inductively coupled plasma mass spectrometer (ICP-MS, Agilent 8900; Santa Clara, CA, USA) for determination of trace elements [30]. As and Hg were determined by atomic fluorescence spectrometer AFS-8220 (Titan Instruments, Beijing, China).

#### 2.2.4. Sequential Extraction Procedure (BCR)

The 12 soil samples were selected and weighed 1.00 g in 100 mL polypropylene centrifuge tubes, and the soil heavy metal forms were analyzed by a modified BCR procedure. Then, 30 mL of 0.11 mol/L CH_3_COOH was used for the exchangeable fraction (F1), 30 mL of 0.1 mol/L NH_2_OH-HCl was used for the reducible fraction (F2); the oxidizable fraction (F3) was extracted with 5 mL of 8.8 mol/L H_2_O_2_ followed by 25 mL of 1 mol/L CHCOONH_4_, residual fraction (F4) was the extraction with HNO_3_-HF-HClO_4_ [31]. The Cd, Co, Cr, Cu, Ni, Pb, and Zn in the extracts of the above steps were determined by ICP-MS (ICP-MS; Agilent 8900; Santa Clara, CA, USA), and As and Hg were determined by atomic fluorescence spectrometer AFS-8220.

#### 2.2.5. Quality Assurance (QA)/Quality Control (QC)

Total organic carbon analyzer can detect TOC concentration detection error of ±1%. The detection limit and detection error of ion chromatography detection of anion and cation are 1 mg/kg and ±5%, respectively. During the test procedures for heavy metals, standard reference samples (GSS-18), blank samples, parallel samples, and study samples were analyzed similarly to control the quality of the entire analytical procedure and ensure comparable detection results. The repeatability of each element of the sample was greater than 95%, and the recovery of the standard sample ranged from 90% to 105% (Table A1, Appendix A).

### 2.3. Contamination and Risk Assessment of Heavy Metals in Soils

#### 2.3.1. Enrichment (*EF*)

The enrichment factor (*EF*) is an essential indicator for evaluating the level of contribution of anthropogenic and natural sources to the elemental content of particulate matter and quantitatively evaluating the degree and source of pollution [32,33], calculated as follows in Equation (1):

(1) $$EF=\frac{C_{i}/C_{ref}{}_{Sample}}{C_{i}/C_{ref}{}_{Brackground}}$$

where *C_i_* is the concentration of soil heavy metal element *i* and *C_ref_* is the reference element content. Al was chosen as the reference element in this study as human activities have a small impact on the content of Al. The background element is the alkaline soil elements in the background value from Chinese soil elements [34]. The enrichment factor *EF* was classified into three levels, *EF* < 1.5 as no contamination, 1.5 ≤ *EF* < 3 as weak contamination, and *EF* ≥ 3 as moderate contamination.

#### 2.3.2. Geo-Accumulation Index

The geological accumulation index (*I_geo_*), commonly referred to as the Muller index [35], is a parameter that distinguishes the impact of anthropogenic activities and is calculated by the following Equation (2):

(2) $$I_{\mathrm{geo}}=\log_{2}\left[C_{i}/k*B_{i}\right]$$

where *C_i_* is the measured content of heavy metal *i*; *k* is the correction index (*k* = 1.5); *B_i_* is the soil background value of heavy metal *i*. According to the ground accumulation index *I_geo_*, the pollution level is divided into 7 levels [36,37], among which, 0~6 levels are: *I_geo_* < 0, no pollution; 0 ≤ *I_geo_* < 1, no pollution to moderate pollution; 1 ≤ *I_geo_* < 2, moderate pollution; 2 ≤ *I_geo_* < 3, moderate pollution to strong pollution; 3 ≤ *I_geo_* < 4, strong pollution; 4 ≤ *I_geo_* < 5, strong pollution to very strong pollution; *I_geo_* ≥ 5, very strong pollution.

#### 2.3.3. Potential Ecological Risk Index

The potential ecological risk index (*RI*) was proposed by Hakanson [38], a Swedish scholar, and based on the physicochemical properties of heavy metals and the interaction with the environment. *RI* evaluated by the grading method with a comparable equivalence property index. Its calculation Formula (3) is as follows:

(3) $$C_{f}^{i}=\frac{C^{i}}{C_{n}^{i}},\;C_{d}=\sum C_{f}^{i},\;E_{r}^{i}=T_{r}^{i}C_{f}^{i},\;RI=\sum E_{r}^{i}$$

where *C_f_^i^* is the contamination factor of heavy metal *i*; *C^i^* is the measured concentration of heavy metal *i*; and *C_n_^i^* is the geochemical background value of heavy metal *i*. *E_r_^i^* and *T_r_^i^* are the ecological risk index and the toxicity coefficient of heavy metal i, respectively. The toxicity coefficient of heavy metal are the values of Zn = 1, Cr = 2, Co = Ni = Cu = Pb = 5, Hg = 40, and Cd = 30. Since the heavy metal species in this study were different from Hakanson [38], the *RI* limits corresponding to each ecological risk level were adjusted according to the heavy metal species and their toxicity coefficients, and the adjusted evaluation criteria were: *RI* < 150 for minor potential ecological risk; 150 ≤ *RI* < 300 for medium potential ecological risk; 300 ≤ *RI* < 600 for strong potential ecological risk; where *RI* < 150 is a slight potential ecological risk; 150 ≤ *RI* < 300 is a medium potential ecological risk; 300 ≤ *RI* < 600 is a strong potential ecological risk; *RI* ≥ 600 is a very strong potential ecological risk [39,40].

#### 2.3.4. Risk Assessment Code

The composition of heavy metals was also used to assess the potential transport transformation and biological effectiveness of heavy metals in soils. The risk assessment index (RAC) method was proposed by Perin et al. [41] based on the binding strength of different fugitive forms of heavy metals and is widely used to evaluate the degree of bioavailability of heavy metals and their environmental risks [42,43,44], which is defined as the mass fraction of the weak acid extracted state of heavy metals and calculated as:

(4) $$\mathrm{RAC}\;=\frac{F1}{F1+F2+F3+F4}$$

The RAC indices can be grouped into the following five categories: RAC < 1%, no risk; 1% < RAC < 10%, low risk; 11% < RAC < 30%, considerable risk; 31% < RAC < 50%, high risk; RAC > 50%, very high risk.

#### 2.3.5. Statistical Analysis

Statistical analyses were performed using the SPSS 19.0 software (IBM, Armonk, NY, USA). Correlation analysis and correlation analysis heat map by origin 2020 (OriginLab Corporation, Northampton, MA, USA). The sampling site map (Figure 1) was drawn using ArcGIS10.2.

## 3. Results

### 3.1. Physicochemical Properties of Surface Soils

**Figure 2:**
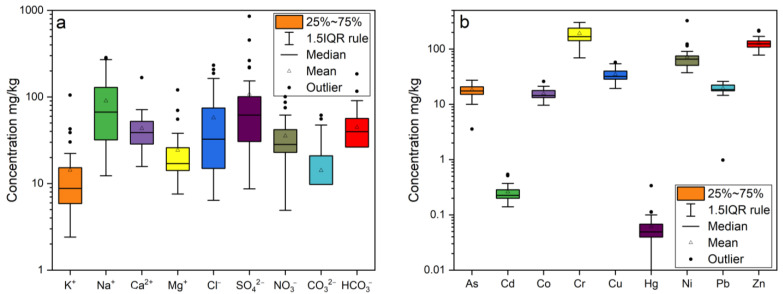
Box plot of anions, cations (**a**) and heavy metal (**b**) concentrations for the 35 surface soils from Hetao Plain in Bayannur.

The EC, pH, K^+^, Na^+^, Ca^2+^, Mg^2+^, Cl^−^, ${\mathrm{SO}}_{4}^{2-}$, ${\mathrm{NO}}_{3}^{-}$, ${\mathrm{CO}}_{3}^{2-}$, and ${\mathrm{HCO}}_{3}^{-}$ in the soil samples collected from the study area are shown in Figure 2a and Table A2 (Appendix A). The EC of surface soils is in the range of 0.16–1.36 mS/cm, averaging 0.46 mS/cm. The pH is in the range of 8.07 to 9.94, averaging 8.63. The main cations in the soluble salt of the surface soil are Na^+^ and Ca^2+^, while the anions were Cl^−^, ${\mathrm{SO}}_{4}^{2-}$, ${\mathrm{CO}}_{3}^{2-}$, and ${\mathrm{HCO}}_{3}^{-}$. The concentrations of K^+^, Na^+^, Ca^2+^, and Mg^2+^ ranged from 2.41 to 105.14, 12.32 to 285.01, 15.76 to 167.16, and 7.56 to 120.23 mg/kg, respectively; the Cl^−^, ${\mathrm{SO}}_{4}^{2-}$, ${\mathrm{NO}}_{3}^{-}$, ${\mathrm{CO}}_{3}^{2-}$, and ${\mathrm{HCO}}_{3}^{-}$ concentrations ranged from 6.41 to 232.34, 8.67 to 854.2, 4.9 to 100.68, 0 to 61.40, and 0 to 184.43 mg/kg, respectively.

The TOC and minerals in the soda soil samples from the study area are shown in Table A3 (Appendix A). The TOC content of the soils in the study area ranged from 0.16–0.89%, with an average of 0.53%. The main minerals are quartz, feldspar, dolomite, calcite, and clay minerals. The clastic minerals are primarily composed of quartz feldspar with contents ranging from 18.5% to 60.3%, 2.3% to 49.9%, with average values of 39.9% and 15.61%, respectively. The carbonate minerals are mainly dolomite and calcite ranging from 6.0% to 56.3%, 0 to 15.7%, with a mean value of 11.9% and 3.5%, and clay minerals ranging from 0 to 42.3%, with a mean value of 25.1%. The concentrations of major elements (Al, Ca, Fe, Mg, and Mn) ranged from 4.41% to 9.57%, 3.34% to 8.17%, 2.32% to 5.56%, 0.90% to 2.50%, 0.04% to 0.11%, and the average values were 7.15%, 6.43%, 3.62%, 1.83%, and 0.07%.

### 3.2. The Concentration of Heavy Metals in Soils

The statistics of heavy metals in the soils of the study area and other agricultural areas in China are shown in Table 1. As shown in Figure 2b, the soil concentration of As, Cd, Co, Cr, Cu, Hg, Ni, Pb, and Zn in the study area ranged (mg/kg) from 3.57–27.33, 0.14–0.52, 9.64–25.92, 69.35–303.90, 19.34–57.61, 0.001–0.33, 37.29–325.6, 0.98–25.95, and 77.8–216.4. The coefficients of the variation in heavy metal concentrations were (%) 27.1, 33.0, 21.8, 31.8, 25.2, 85.4, 65.2, 22.6, and 21.7, indicating that the content of Hg and Ni in the soil of the Bayannur was highly variable, and the intensity of variation in Cr and Cd was moderate (Table A4, Appendix A). Apart from Cr, the levels of Ni, As, Cd, Co, Cu, Hg, Pb, Zn, and other heavy metals were less than the heavy metal levels in agricultural soils of Bayan Obo, Lianyuan, Pearl River, Guanting Reservoir, and Lijiang River.

### 3.3. Geochemical Fractionations of Heavy Metals

The BCR extraction method classifies soil heavy metals into four chemical forms: acid extractable; reducible (Fe-Mn oxide bound); oxidizable (organic bound); and residue. The acid extractable form covers the exchangeable and carbonate-bound forms, which are highly mobile and can be directly absorbed and used by organisms. Heavy metals in the residue form exist mainly in the crystalline matrix of primary and secondary minerals and silicates, which are the most stable and cannot be easily released under normal natural conditions. The composition of heavy metals in soils is closely related to the migration, transformation, cycling processes, and environmental impacts of heavy metals in the surface soil. Therefore, the composition of heavy metals is more likely to accurately assess the environmental impact of soil heavy metals as opposed to the assessment of heavy metal concentrations in soil.

**Figure 3:**
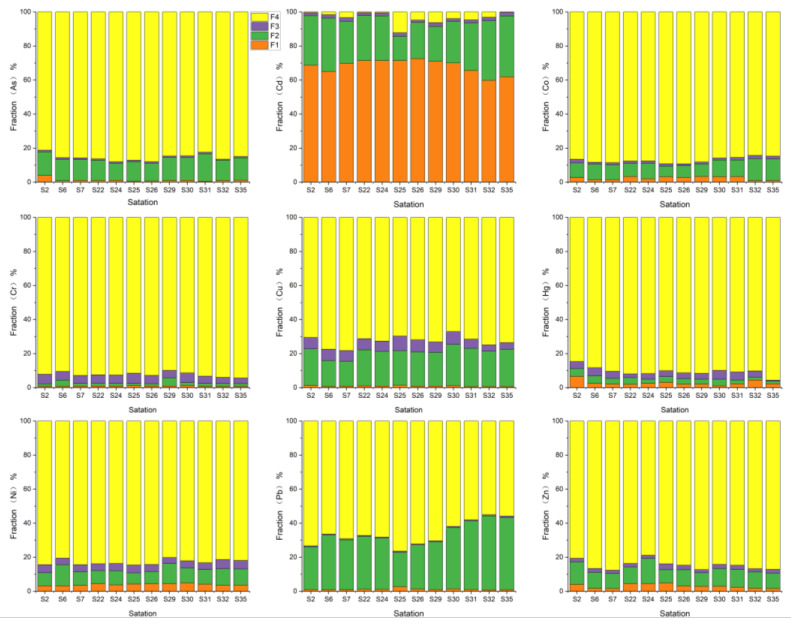
Percentage distribution of heavy metals: the exchangeable fraction (F1), the reducible fraction (F2), the oxidizable fraction (F3), and residual fraction (F4) at 19 stations.

The results of the heavy metal composition of these soils showed that the residual fraction (F1) of As, Co, Cr, Hg, Ni, and Zn was higher than 85% of the heavy metal of the soil, indicating that these heavy metals are mainly embedded in the mineral lattice and cannot be easily migrated for plant uptake. The content of the oxidizable fraction (F2) of soil heavy metals is low. The reducible fraction (F2) of Cd, Cu, and Pb in the soil is relatively high, accounting for about 20%, followed by As, Co, Ni, and Zn, accounting for about 10%, and Cr and Hg are low, accounting for less than 5%. The weakly acid-extracted fraction (F1) of Cd in soils is higher than all other heavy metals, accounting for more than 50% (Figure 3). Since the weakly acid-extracted fraction of soil heavy metals has stronger mobility and transformation [50], it is easily absorbed by plants, causing greater ecological impact and harm to human health. Therefore, the biological effectiveness of Cd under weakly acidic conditions is great, and it is easily absorbed by plants.

### 3.4. Pollution and Risk Assessment

#### 3.4.1. *EF* and *I_geo_*

**Figure 4:**
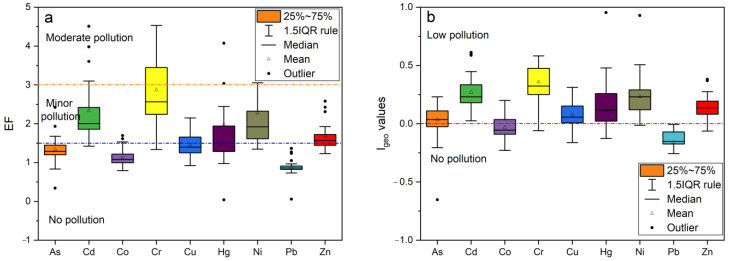
*EF* (**a**) and *I_geo_* (**b**) values of heavy metals for the 35 surface soils from Hetao Plain in Bayannur.

The *EF*s of the nine heavy metals in the soils were in the order of Cr (2.87) > Cd (2.34) > Ni (2.27) > Hg (1.97) > Zn (1.68) > Cu (1.45) > As (1.33) > Co (1.13) > Pb (0.87). The *EF*s of As, Co, Cu, and Pb were less than 1.5, indicating no contamination, and the *EF*s of Cd, Cr, Hg, Ni, and Zn had *EF* values between 1.5 and 3, which are moderately contaminated. According to this standard As, Co, Pb is no pollution; Cd, Ni, Hg, Zn, Cu is no pollution–minor pollution; Cr is minor pollution–moderate pollution (Figure 4a). Figure 4b shows the *I_geo_* values of the nine heavy metals in these soils in the order of Cr (0.36) > Cd (0.27) > Ni (0.23) > Zn (0.14) > Hg (0.11) > Cu (0.08) > As (0.03) > Co (−0.03) > Pb (−0.16). The *I_geo_* values of Co and Pb are negative, indicating no contamination. The range of *I_geo_* values of Cr, Cd, Ni, Zn, Hg, Cu, and As are between 0 and 1, indicating that the study area is low contamination.

#### 3.4.2. Potential Ecological Risk Index

**Figure 5:**
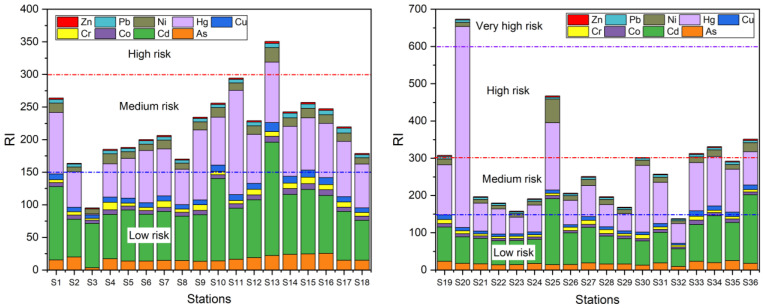
*RI* values of heavy metals in the 35 surface soils from Hetao Plain in Bayannur.

**Figure 6:**
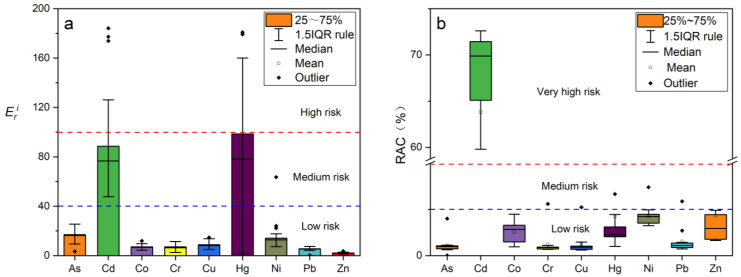
$E_{r}^{i}$ (**a**) and RAC (**b**) values of heavy metals in the 35 surface soils from Hetao Plain in Bayannur.

In this work, the summation of potential ecological risk coefficients of heavy metal contaminants for 36 soil samples showed that most of the samples were of medium ecological risk. Only one sample showed very high ecological risk, two samples showed high ecological risk, and two samples showed low ecological risk (Figure 5). The values of potential ecological risk ($E_{r}^{i}$) for individual heavy metals in the soils of the study area decreased in the following order: Hg > Cd > As > Ni > Cu > Cr > Co > Pb > Zn (Figure 6a). Cd and Hg responded to moderate ecological risk, while As, Ni, Cu, Cr, Co, Pb, and Zn all belonged to slight ecological risk, indicating that most of the heavy metals had low ecological risk.

#### 3.4.3. Risk Assessment Code

The risk assessment code (RAC) of the 12 selected heavy metals in the study area are shown in Figure 6b. The RAC results show that As, Co, Cr, Cu, Hg, Ni, Pb, and Zn pose low or no risk to the soda soils (RAC < 10%), and these heavy metals have low mobility and bioavailability. Cd presents a high risk to soils (RAC > 60%). The results above show that the different methods for estimating the ecological risk of heavy metals are inconsistent, mainly due to differences in the purpose of assessing pollution risk. *EF* and *I_geo_* focus on the enrichment and accumulation of heavy metals relative to background soil, while *RI* assesses ecological risk based on the biotoxicity of different heavy metals, and RAC emphasizes the mobility of heavy metals, i.e., the components that can be taken up by plants. Therefore, it could be concluded that most of the soils in the study area have low levels of heavy metals and are non-contaminated or mildly contaminated. However, as the most biotoxic Hg and Cd, the soil in the study area has a high ecological risk. Owing to the contribution of these two heavy metals, the *RI* of the sampling sites in the study area exhibits a medium risk–high risk. It is also notable that low levels of Cd are highly biotoxic and mobile, which should be passivated or adsorbed in various conditions at the pH < 7.

## 4. Discussion

### 4.1. Source Analysis of Heavy Metals in Surface Soils

**Figure 7:**
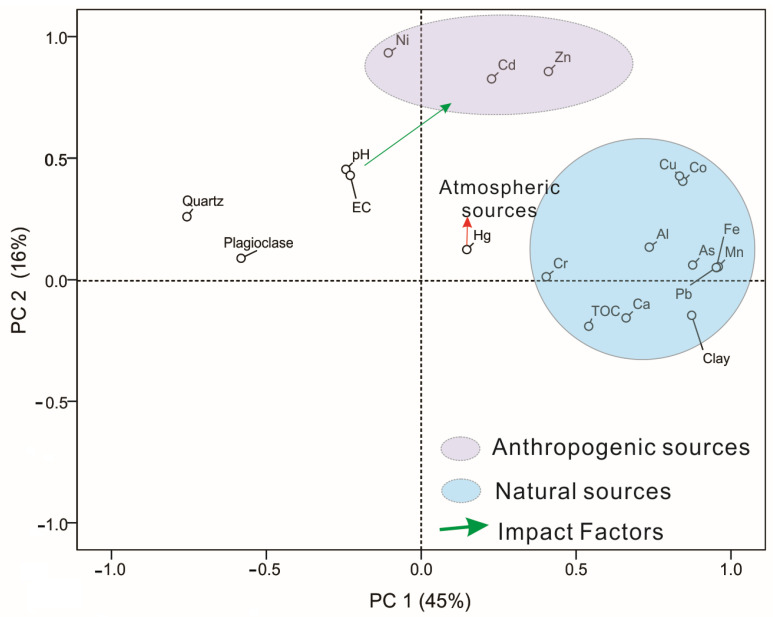
PCA for the nine heavy metals, pH EC, and elements in surface soils from Hetao Plain in Bayannur.

For the purpose of identifying the sources of heavy metals and their influencing factors, principal component analysis (PCA) and correlation analysis were used for As, Cd, Co, Cr, Cu, Hg, Ni, Pb, Zn, pH, EC, Quartz, Plagioclase, and clay (Figure 7). The PCA results passed the Bartlett sphericity tests (*p* < 0.001), indicating that the application of PCA was appropriate. The first principal component (PC1) and the second principal component (PC2) of the soil samples that explained the variance accounted for 45% and 16% of the total variance, respectively. PC1 was significantly positively loaded with Al (0.74), Fe (0.96), Ca (0.66), Mn (0.96), As (0.88), Co (0.85), Cr (0.41), Cu (0.84), Pb (0.95), clay (0.88), and TOC (0.88) and negatively loaded with Quartz (−0.76) and Plagioclase (−0.58). PC2 was significantly positively loaded with Cd (0.83), Ni (0.93), and Zn (0.86).

**Figure 8:**
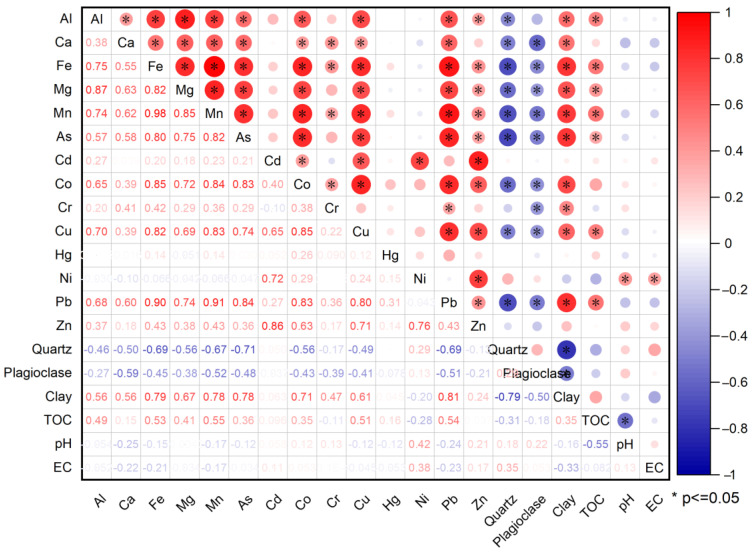
The hot map of correlation between soil heavy metals and influencing factors for the 35 surface soils from Hetao Plain in Bayannur.

Combined with the composition of heavy metal in the soda soils, it is concluded that there are three sources of heavy metals as follows: (1) As, Co, Cr, Cu, and Pb have a highly positive correlation with clay minerals (Figure 8), Fe, and Mn, and are mainly distributed in the soil in the residue fraction, which principally comes from the natural accumulation of sediments from the alluvial plains. The sediment transport not only contributes to the particle size-sorting, but also promotes the adsorption of these trace elements by fine-grained clay minerals and organic matter. The As distribution of soils and groundwater in the Hetao Plain shows that the heavy metal content decreases with distance from the upstream Yinshan mountain front [51], which also confirms the origin of these metals from the matrix sediments; (2) Ni, Cd, and Zn mainly originate from anthropogenic pollution-related irrigation, fertilizers, and pesticides, among which the formation of Cd is mainly in the acid-soluble state, indicating that it may be anthropogenic sewage discharge and form carbonate precipitation after entering alkaline water, considering the high content of Ni and Zn in the soil, most of them form insoluble residue fraction after entering the environment; (3) The source of Hg in soil shows that it is obviously unknown from the above two, and studies have shown that atmospheric long-range transport is the main form of Hg migration, therefore, it is believed that the source of Hg in the soil in the study area is atmospheric deposition, especially by snow in winter [52,53].

### 4.2. Influencing Factors for the Accumulations of Heavy Metals

The bioavailability, toxicity, and mobility of heavy metals were affected by soil or water pH, soluble salt composition, organic matter, and clay minerals. A fine-grained fraction of minerals plays a significant role in the quality of water and sediment, especially in the adsorption of metals [54], and even the degree of metals’ accumulation in biological tissues. The fine-grained fraction in sediment, compared to coarse particles, generally contains greater concentrations of trace metals because of their larger surface area and higher cation exchange capability [55,56,57,58,59,60,61] As, Co, Cr, Cu, and Pb in the soda soils of the study area are mainly of natural origin, and these soil organic matter, clay minerals, and metal elements have distinct sediment properties. The value of pH is used to weigh the acidity or alkalinity in sediment or water column and strongly influence the solubility of trace metals. High pH values promote adsorption and precipitation while low pH can actually weaken the strength of metal association and impede the retention of metals by sediments [62,63]. In addition, the increase in salinity was related to an enhancement in the content of major cations (e.g., Na, K, Ca, Mg) that compete for the sorption sites with heavy metals and decreased the binding of metals to humic acids [64].

In the process of transporting and sorting the clastic particles from the mountain front to the downstream, the fine-grained sediments are gradually enriched in the downstream area, and these metal elements and organic matter are adsorbed with the clay mineral surface and interlayer, forming a complex of organic matter, metal elements, and clay minerals. The salinity of soda soils in the study area has a good positive correlation with pH, so the influence of these two factors on the content of soil heavy metals is consistent. For heavy metals of anthropogenic origin, the increase in salinity and pH favored the precipitation of metal elements in the water column, and the lower content of Cd combined with ${\mathrm{CO}}_{3}^{2-}$ or OH^−^ to form exchangeable and reduced state components, while the higher content of Ni and Zn was mostly chelated by organic matter and clay minerals, except for a small amount combined with ${\mathrm{CO}}_{3}^{2-}$ or OH^−^.

## 5. Conclusions

In this paper, the pH, salinity, and mineral composition of the agricultural soil in Hetao Plain of Bayannur were first analyzed. As a typical sodic soil, it has a pH greater than 8, and the cations in the soluble salt of the surface soil are Na^+^, K^+^, and Ca^2+^, while the anions were Cl^−^ and ${\mathrm{SO}}_{4}^{2-}$. ${\mathrm{CO}}_{3}^{2-}$, and ${\mathrm{HCO}}_{3}^{-}$, and the soil have a high content of calcite, dolomite, and clay. The concentrations of As, Cd, Co, Cr, Cu, Hg, Ni, Pb, and Zn in the agricultural soil was slightly lower or equal to the soils or sediments from other regions of China. The *EF* and *I_geo_* indicated that these heavy elements are non-contaminating or of low contamination levels, with a relatively low degree of anthropogenic contamination. *RI* and the Risk Assessment Code indicated Cd and Hg responded to moderate ecological risk, while As, Ni, Cu, Cr, Co, Pb, and Zn all belonged to a slight ecological risk. In particular, since Cd is a mainly exchangeable and reducible fraction in the study area, appropriate measures should be taken for passivation or adsorption under an alkaline environment.

The compositions of heavy metals, principal component analysis, and correlation analysis showed that these heavy metals come from three sources. As, Co, Cr, Cu, and Pb mainly came from the natural accumulation of sediments from the alluvial plains. The contents of these metal elements in soil are controlled by particle size-sorting effect and usually exist as residues combined with clay minerals and organic matter. Ni, Cd, and Zn are mainly derived from anthropogenic discharge-related irrigation, fertilizers, and pesticide application. Due to the synergistic influence of pH and salinity, these metal elements precipitate rapidly after entering water; Cd and ${\mathrm{CO}}_{3}^{2-}\;$or OH^−^ combine to form the weak acid exchangeable fraction or reduced fraction, while Ni and Zn are adsorbed by clay minerals to form the residue. Hg originated mainly from a source of atmospheric fallout, and probably from the recorded winter snowfall in Hetao Plain of Bayannur. The Hg levels in these soda soils do not appear to be related to clay minerals, pH, or salinity.

## Figures and Tables

**Table 1:** The ranges of the heavy metal content of soil (sediment) from Bayannur and other regions in China (mg/kg).

HM	Bayannur	Bayan Obo	Lianyuan	Pearl River	Guanting Reservoir	Lijiang River
As	3.57–27.33	7.70–22.73	0.78–512.05	/	3.06–10.90	9.97–36.44
Cd	0.14–0.52	0.08–5.28	0.05–8.71	0–1.76	0.39–1.20	0.16–4.41
Co	9.64–25.92	/	/	0.22–39.90	/	4.50–15.38
Cr	69.35–303.9	28.95–102.64	21.49–206.20	13.3–144.00	16.78–59.40	24.38–95.38
Cu	19.34–57.61	11.73–72.29	7.92–719.60	1.41–44.00	2.86–64.40	9.38–102.75
Hg	0.001–0.33	0.01–0.71	1.20–3601.10	/	/	0.08–2.13
Ni	37.29–325.6	/	/	3.55–78.60	5.95–33.30	11.63–37.13
Pb	0.98–25.95	15.94–502.35	8.69–744.70	7.74–54.70	1.74–165.00	17.88–171.75
Zn	77.8–216.4	/	21.13–1112.00	14.80–110.00	22.99–109.00	53.63–258.00
	This study	Wang et al., 2012 [45]	Liang et al., 2017 [46]	Wong et al., 2002 [47]	Luo et al., 2007 [48]	Xiao et al., 2021 [49]

HM = Heavy metals.

## Data Availability

The data are contained within the article.

## Appendix A

**Table A1:** The results of heavy metals in standard reference samples (GSS-18).

Sample	GSS-18	GSS-18	GSS-18	GSS-18	GSS-18	Actual Value	Detection Limit (mg/kg)
Cr	54.653	56.212	56.75	53.957	55.721	55 ± 2	0.005
Ni	25.471	24.53	24.686	26.243	25.796	25 ± 1	0.005
Cu	19.981	19.574	29.889	19.532	19.663	19.5 ± 0.5	0.01
Zn	62.627	654966	63.432	62.653	63.519	63 ± 2	0.02
As	10.289	10.688	11.164	10.667	11.106	10.7 ± 0.5	0.05
Cd	0.146	0.159	0.148	0.158	0.141	0.15 ± 0.01	0.01
Pb	19.985	20.817	19.972	19.858	20.667	20 ± 1	0.005
Co	9.995	10.468	10.254	9.965	10.462	10.2 ± 0.3	0.005
Hg	0.016	0.014	0.016	0.018	0.013	0.015 ± 0.003	0.005

**Table A2:** Maximum, minimum, and mean values of anions and cations, pH, and EC of soil in Bayannur City.

	K^+^	Na^+^	Ca^2+^	Mg^2+^	Cl^−^	${SO}_{4}^{2-}$	${NO}_{3}^{-}$	${CO}_{3}^{2-}$	${HCO}_{3}^{-}$	pH	EC
min	2.41	12.32	15.76	7.56	6.41	8.67	4.90	ND.	0.00	8.07	165.15
max	105.14	285.01	167.16	120.23	232.34	854.20	100.68	61.40	184.43	9.94	1358.00
mean	14.26	89.87	43.27	24.30	57.38	107.65	35.44	14.16	44.64	8.63	458.41

Note: “ND.” means not detected.

**Table A3:** Maximum, minimum, and mean values of TOC and main minerals of soil in Bayannur City.

	TOC	Quartz	Plagioclase	Calcite	Dolomite	Clay
min	0.16	18.50	2.30	6.00	ND.	ND.
max	0.89	60.30	49.90	56.30	15.70	42.30
mean	0.53	39.92	15.61	11.98	3.51	25.08

Note: “ND.” means not detected.

**Table A4:** Maximum, minimum, mean, and CV values of heavy metals in soil from Bayannur City.

	As	Cd	Co	Cr	Cu	Hg	Ni	Pb	Zn
min	3.57	0.14	9.64	69.35	19.34	ND	37.29	0.98	77.80
max	27.33	0.52	25.92	303.90	57.61	0.34	325.60	25.95	216.40
mean	18.13	0.25	15.50	191.86	34.19	0.06	71.06	19.31	124.73
CV (100%)	27.06	33.03	21.78	31.85	25.20	85.45	65.25	22.57	21.75

Note: “ND.” means not detected.
